# Supplementary material for: Hypothermia protects against ventilator-induced lung injury by limiting IL-1β release and NETs formation
Source: eLife. 2025 Jun 24;14:RP101990. doi: 10.7554/eLife.101990 (PMC12187133; doi:10.7554/eLife.101990)
Supplement: Figure 7—source data 1. [file elife-101990-fig7-data1.pdf]

Sup., IL-1β

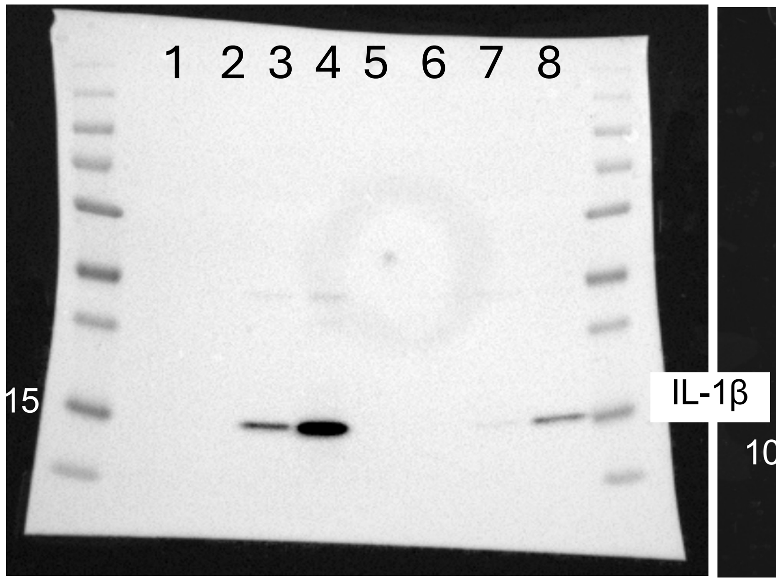

Sup., Casp1

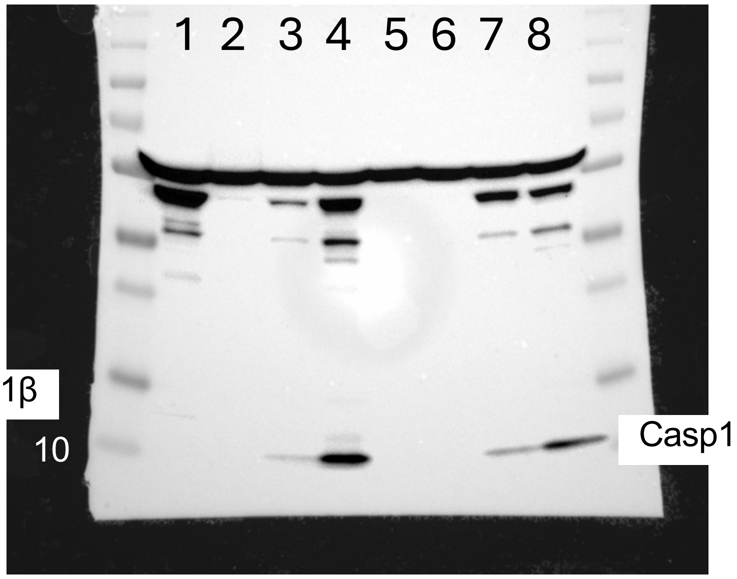

Lysate, IL-1β

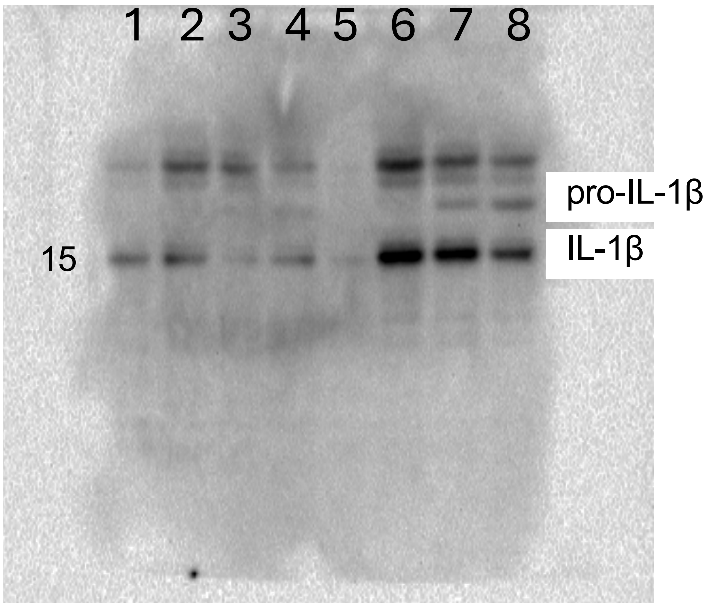

Lysate, Ponceau S

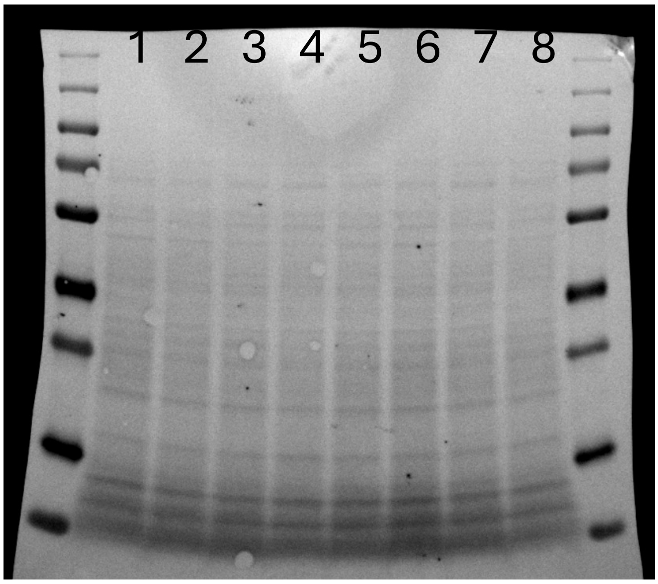

1. 37˚C, Medium
2. 37˚C, LPS
3. 37˚C, LPS+ATP
4. 37˚C, LPS+NIG
5. 32˚C, Medium
6. 32˚C, LPS
7. 32˚C, LPS+ATP
8. 32˚C, LPS+NIG

Figure 7-source data 1 : Figure 7E

Sup., GSDMD

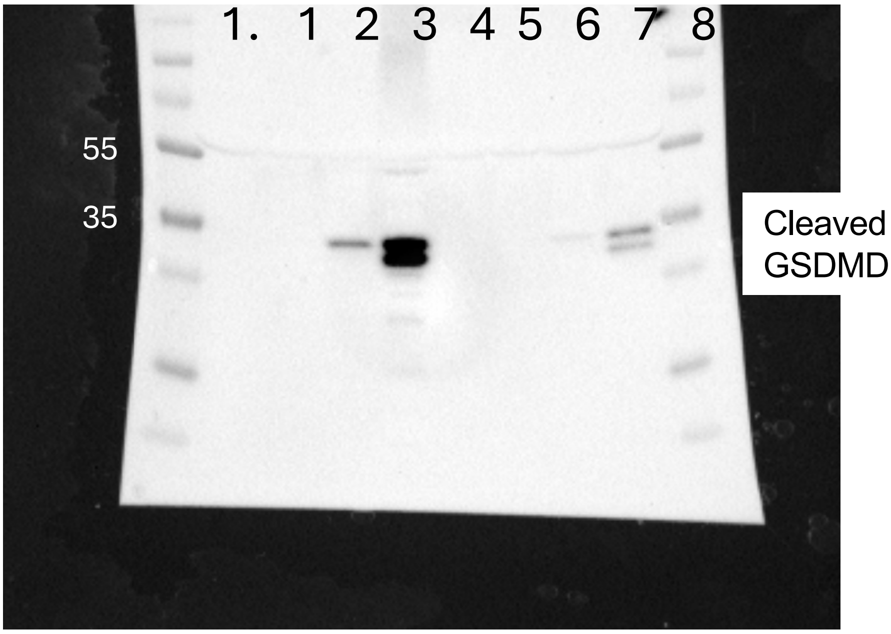

Lysate, GSDMD

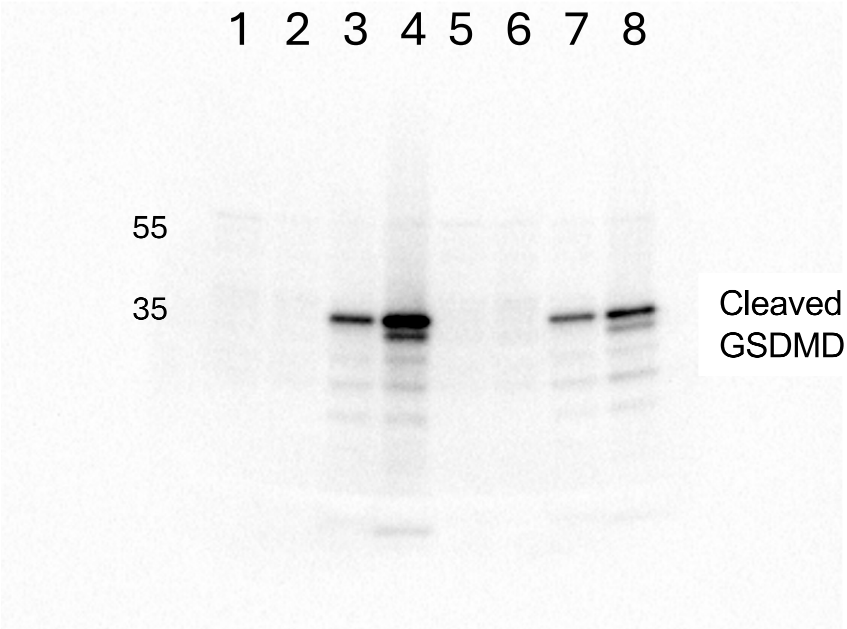

1. 37˚C, Medium
2. 37˚C, LPS
3. 37˚C, LPS+ATP
4. 37˚C, LPS+NIG
5. 32˚C, Medium
6. 32˚C, LPS
7. 32˚C, LPS+ATP
8. 32˚C, LPS+NIG

Figure 7-source data 1 : Figure 7H
