# Supplementary figures and images for: Hypothermia protects against ventilator-induced lung injury by limiting IL-1β release and NETs formation
Source: eLife. 2025 Jun 24;14:RP101990. doi: 10.7554/eLife.101990 (PMC12187133; doi:10.7554/eLife.101990)

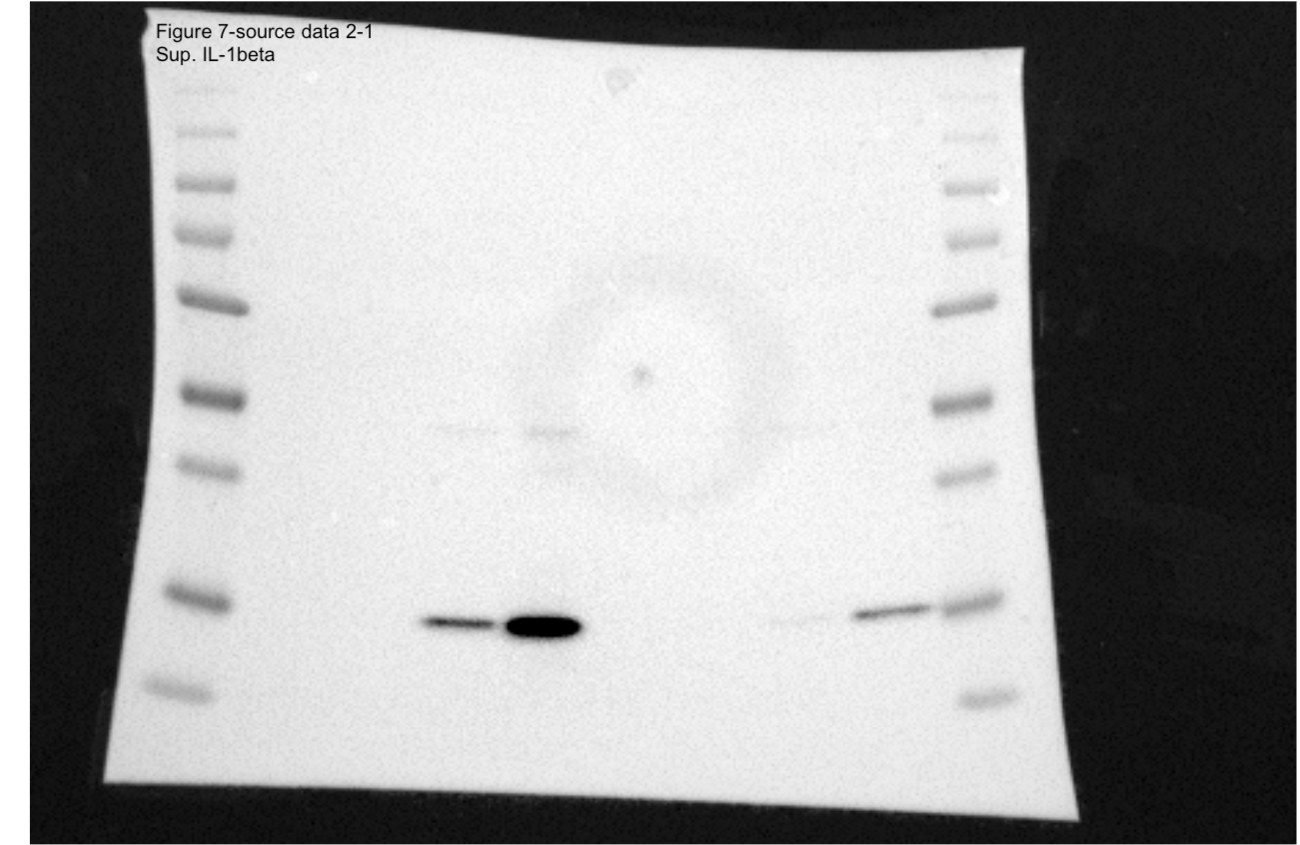

Supplement: Figure 7—source data 2. [file elife-101990-fig7-data2.zip › Figure 7-sourse data 2 /Figure 7-source data 2-1 Sup. IL-1b.tiff]

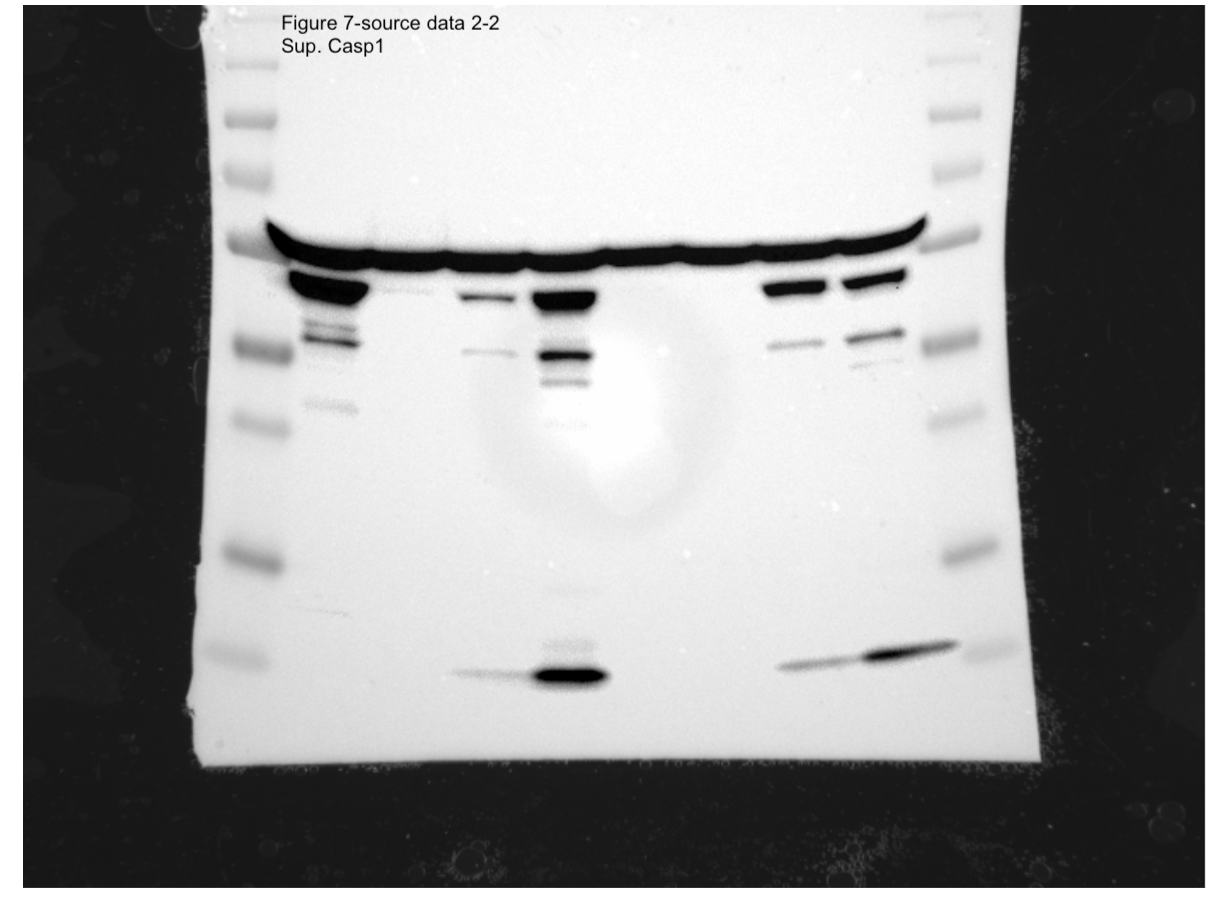

Supplement: Figure 7—source data 2. [file elife-101990-fig7-data2.zip › Figure 7-sourse data 2 /Figure 7-source data 2-2 Sup. Casp1.tiff]

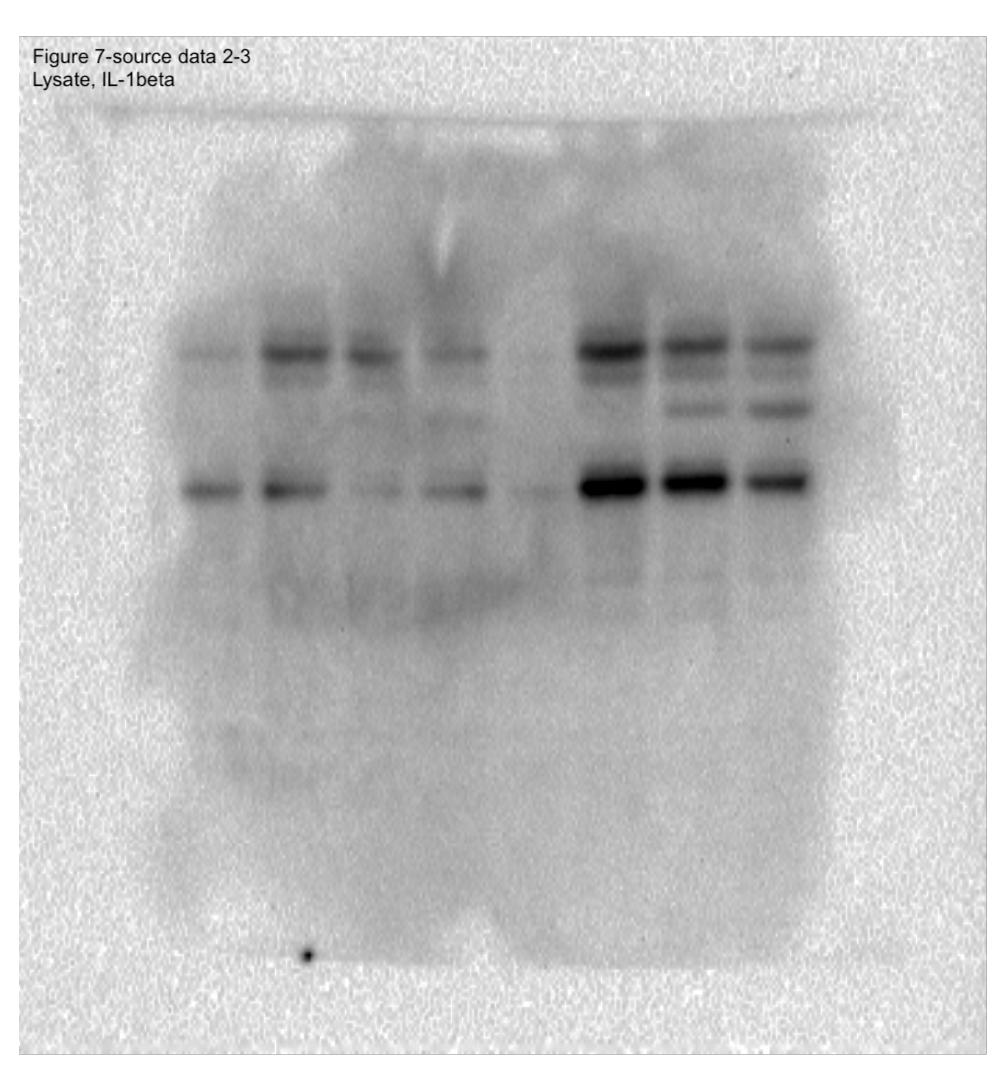

Supplement: Figure 7—source data 2. [file elife-101990-fig7-data2.zip › Figure 7-sourse data 2 /Figure 7-source data 2-3 Lysate IL-1b.tiff]

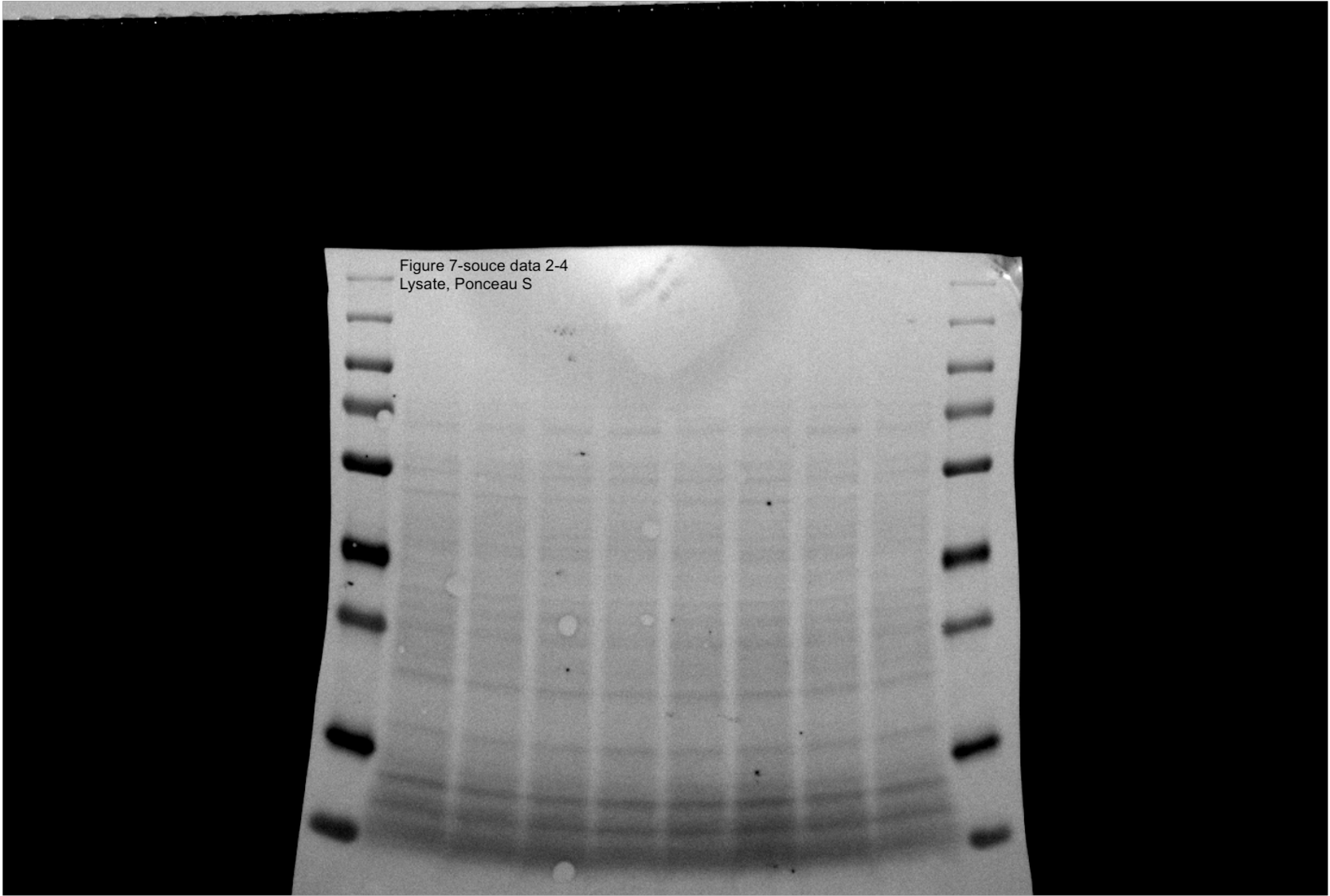

Supplement: Figure 7—source data 2. [file elife-101990-fig7-data2.zip › Figure 7-sourse data 2 /Figure 7-source data 2-4 Lysate Ponceau S.tiff]

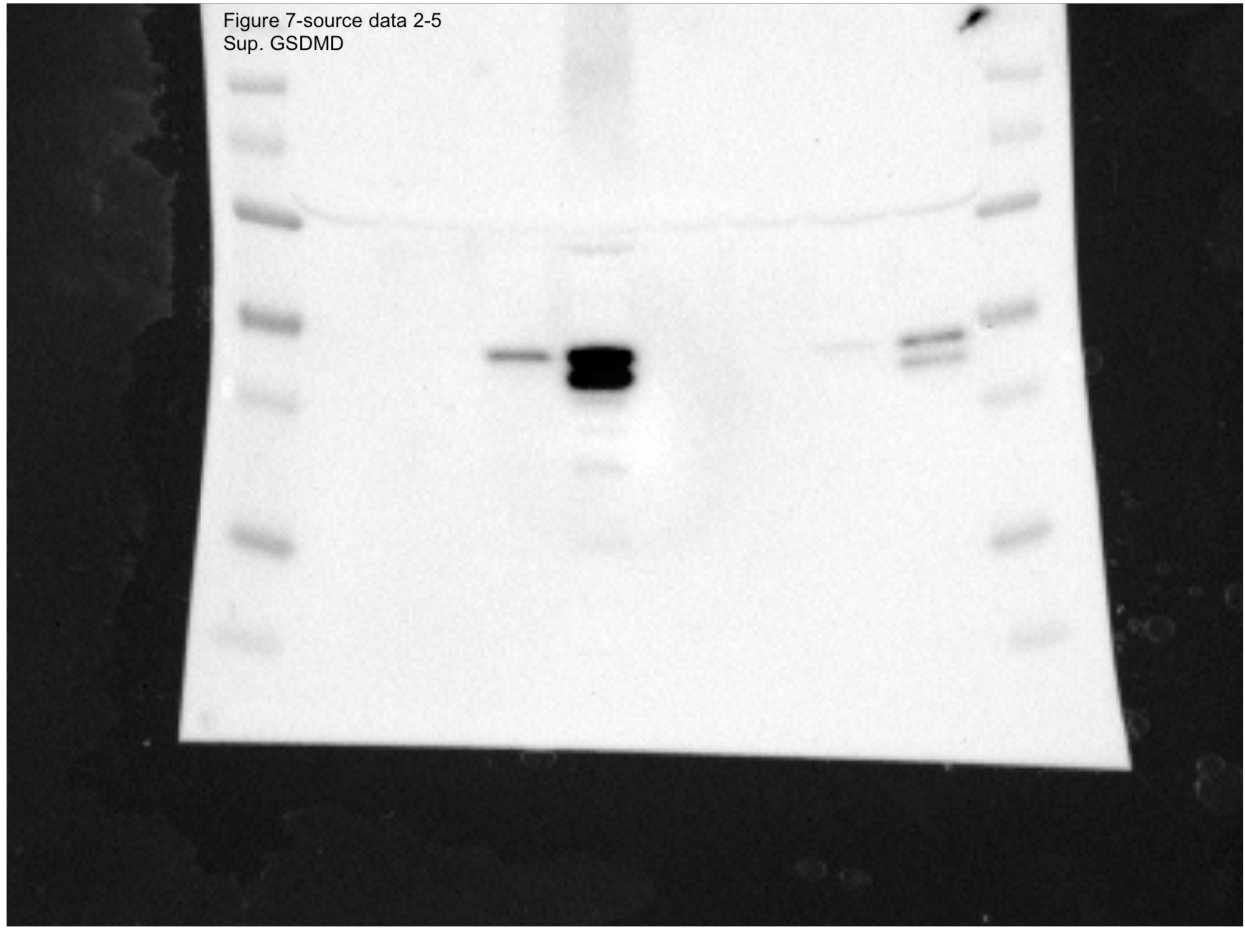

Supplement: Figure 7—source data 2. [file elife-101990-fig7-data2.zip › Figure 7-sourse data 2 /Figure 7-source data 2-5 Sup. GSDMD.tiff]

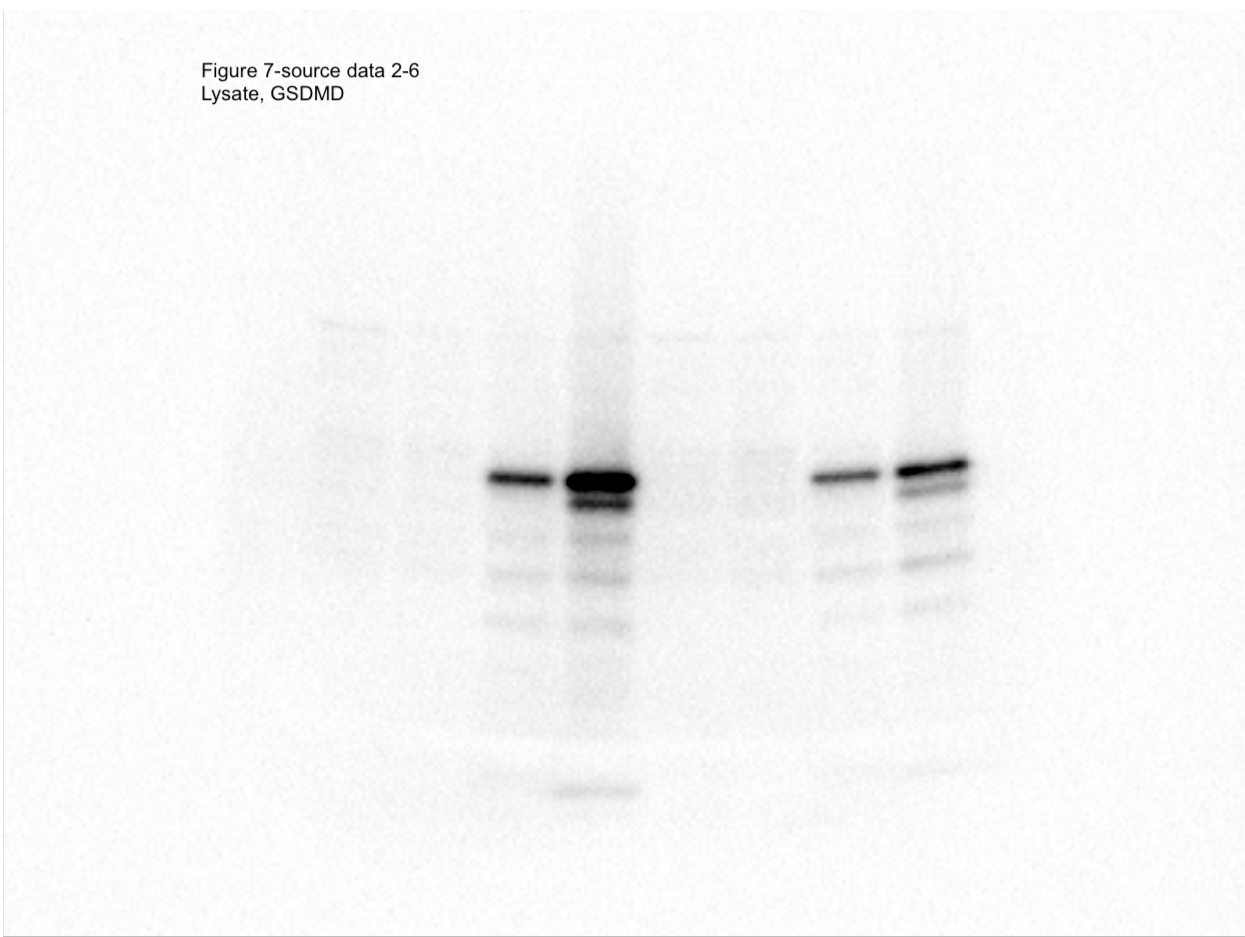

Supplement: Figure 7—source data 2. [file elife-101990-fig7-data2.zip › Figure 7-sourse data 2 /Figure 7-source data 2-6 Lysate GSDMD.tiff]
